# Ins and outs of HSCs and VEGF-induced vascular remodeling: Toward improved HSC mobilization and engraftment

**DOI:** 10.1016/j.stemcr.2025.102569

**Published:** 2025-07-08

**Authors:** Madeline J. Caduc, Simón Méndez-Ferrer

**Affiliations:** 1 Department of Medical Physiology and Biophysics, University of Seville, Seville, Spain; 2 Institute of Biomedicine of Sevilla – IBiS (Hospital Universitario Virgen del Rocío/CSIC/Universidad de Sevilla), Seville, Spain; 3 Cambridge Stem Cell Institute, University of Cambridge, Cambridge, UK; 4 Department of Haematology, University of Cambridge, Cambridge, UK; 5 NHS Blood and Transplant, Cambridge, UK

## Main text

**Figure 1:**
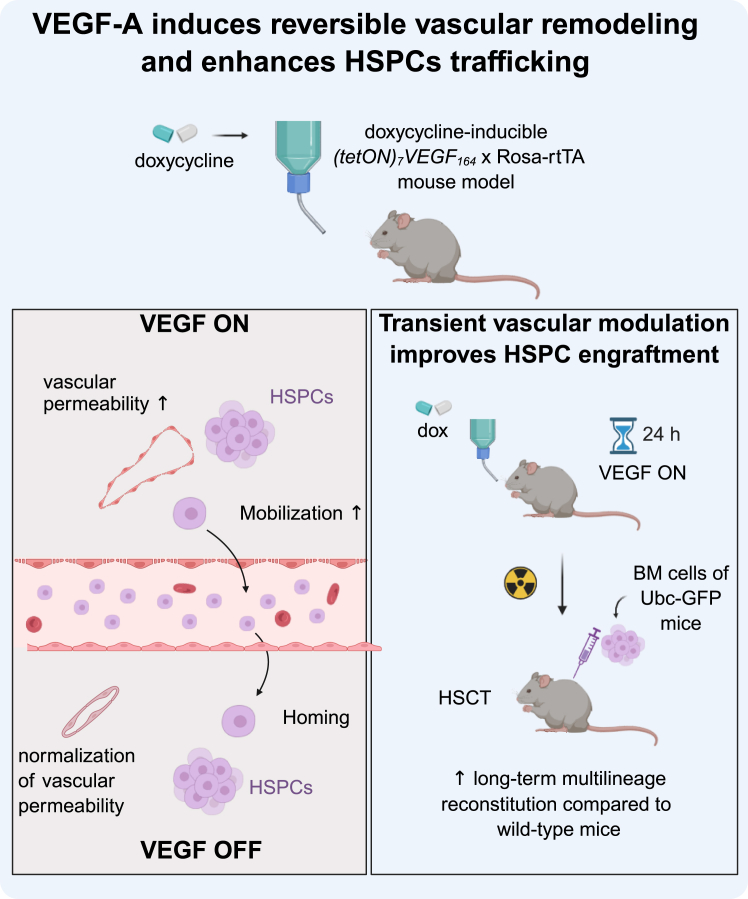
Proposed VEGF-A-based HSC mobilization strategy Forsberg and colleagues (Smith-Berdan et al.) demonstrate that transient VEGF exposure enables safe and rapid HSPC mobilization and enhances engraftment by conditioning the perivascular niches, offering a novel strategy to improve hematopoietic stem cell transplantation (HSCT) outcomes; created with BioRender.

Mobilization of hematopoietic stem cells (HSCs) is essential for successful stem cell transplantation but remains insufficient in a significant subset of patients. In this issue, Smith-Berdan et al. present a novel strategy using transient vascular endothelial growth factor (VEGF)-A overexpression to induce bone marrow (BM) vascular permeability and promote robust, reversible HSC mobilization. This tunable system enhances HSC egress, long-term engraftment, and donor chimerism while preserving vascular integrity (Figure 1). The findings underscore the potential of temporally controlled vascular modulation to optimize HSC trafficking, mobilization, and engraftment, offering a promising adjunct to current transplantation protocols and regenerative therapies.

The mobilization of HSCs from their BM niches allows for their non-invasive harvesting (apheresis) from peripheral circulation for HSC transplantation (HSCT) procedures; yet, the HSC yield obtained upon mobilization is insufficient in up to 30% individuals, prompting the search for new mobilizers. In this issue of *Stem Cell Reports*, an alternative approach for HSC mobilization is presented and consists of timed induction of vascular permeability via VEGF (Smith-Berdan et al.).

Mobilization of HSCs from BM to peripheral blood is a cornerstone of HSCT. While granulocyte-colony stimulating factor (G-CSF) remains the standard mobilizing agent (Drize et al., 1995), a significant proportion of patients either fail to respond adequately or are ineligible due to disease-specific contraindications (Worel et al., 2024). Mobilization failure results from multiple factors, including toxicity of standard mobilization regimens (e.g., G-CSF + cyclophosphamide); the effects of novel induction therapies; and patient-specific variables, such as advanced age, prior cytotoxic treatments, and metabolic comorbidities (Cottler-Fox et al., 2003; Worel et al., 2024). Thus, the need remains for innovative strategies that improve the HSC mobilization efficiency and stem cell yield available following apheresis.

To overcome the limitations of conventional mobilization strategies, alternative agents including the CXCR4 antagonist plerixafor (AMD3100) (Broxmeyer et al., 2005) have shown improved safety and efficacy by disrupting CXCL12-mediated stem cell homing (Cottler-Fox et al., 2003; Worel et al., 2024). Given the critical role of the BM microenvironment in regulating HSPC fate and homing, alternative regimens targeting HSPC-niche interactions are under investigation. For example, a dual α4β1/α9β1 integrin inhibitor that disrupts HSPC interactions with the endosteal niche has demonstrated rapid and effective mobilization, particularly in combination with plerixafor, and may enhance engraftment potential compared to current standard regimens (Cao et al., 2016).

The fenestrated endothelium of BM sinusoids allows for the transmigration of HSCs into/from peripheral circulation; in contrast, the endosteal niche is enriched in less permeable vessels (arterioles and transitional vessels and capillaries) and has been postulated to preserve HSC quiescence under proliferative stress (Mendez-Ferrer et al., 2020). Beyond serving as a conduit for mature hematopoietic cells, the perisinusoidal niche plays a critical role in steady-state hematopoiesis and permits the release of hematopoietic cells into the circulation for further maturation in secondary lymphoid organs or in situations of extramedullary hematopoiesis, when the BM niche is compromised (Mendez-Ferrer et al., 2020). The adhesion molecules anchoring HSCs to these endosteal and non-endosteal perivascular niches (e.g., VLA-4/VCAM-1 axis) and the chemokines directing HSC migration (such as CXCL12) have long been investigated to optimize HSC mobilization strategies (Cottler-Fox et al., 2003; Worel et al., 2024). For example, the VLA-4 antagonist BIO5192 and, more recently, the pegylated VLA-4 inhibitor SLU-2609, disrupt the VCAM-1/VLA-4 axis and promote robust mobilization comparable to G-CSF, either alone or in combination with plerixafor (Cancilla et al., 2024). Together with previous studies demonstrating the importance of vascular integrity in HSPC trafficking, these findings underscore the perivascular niche modulation as a promising strategy to enhance HSPC mobilization.

In this issue of *Stem Cell Reports*, the authors (Smith-Berdan et al.) investigate the use of VEGF-A as a candidate HSC mobilizer (Zhu et al., 2014). Using a doxycycline-inducible VEGF-A-overexpressing mouse model (VEGF^OE^), the authors demonstrate that transient VEGF-A upregulation rapidly induces BM sinusoidal permeability, leading to robust and reversible HSPC egress into the circulation. They demonstrate that VEGF-induced vascular remodeling is a tunable and biologically potent trigger of HSC mobilization. As early as 8 h after doxycycline induction, HSCs (Lin^low^/CD27^+^/c-Kit^+^/Sca-1^+^/Flk2^−^) and multipotent progenitors (MPPs; Lin^low^/CD27^+^/c-Kit^+^/Sca-1^+^/Flk2^+^) were mobilized to the peripheral blood, peaking at 24 h and amounting after 48 h to ∼25-fold-increased circulating HSCs, compared with wild-type controls. While precise quantification of HSCs would require additional long-term transplantations using limiting dilutions, the overall data demonstrate that VEGF-A^OE^-mobilized HSPCs exhibited robust long-term engraftment in this study.

By leveraging the titratable doxycycline on/off system, Smith-Berdan and colleagues effectively decouple transient VEGF-A-induced increases in vascular permeability from potential long-term disruption of the hematopoietic niche. A key strength of this approach is the clear demonstration that VEGF-A-driven vascular and hematopoietic changes are reversible. Following doxycycline withdrawal, mobilized HSPCs efficiently returned to the BM, and the previously elevated vascular permeability returned to baseline levels within a few days. In contrast, prolonged VEGF-A overexpression proved to be harmful: extended vascular leakage led to 50% mortality, consistent with prior studies employing adenoviral VEGF delivery models (Hattori et al., 2001). As previously mentioned, the peak of HSC egress was observed as early as 24 h after VEGF-A induction, suggesting a narrow window during which vascular modulation can be harnessed for mobilization without inducing significant tissue damage. These findings highlight the critical importance of tightly controlled, time-limited modulation of vascular permeability when designing clinical strategies for HSC mobilization or niche conditioning.

During transplantation, a significant proportion of mobilized HSPCs are lost to peripheral organs and fail to home or engraft within the BM, highlighting the need not only for improved mobilization strategies but also for enhancing BM homing and engraftment efficiency. Despite its clinical relevance, the precise mechanisms governing HSPC homing to the BM niche remain incompletely understood. Different strategies to improve homing efficiency are under investigation, including modulation of chemokines receptors and gene editing approaches to enhance their responsiveness to niche-derived signals. Another promising avenue involves modulating the BM microenvironment itself, for example, through conditioning regimens that preserve non-hematopoietic niche components and improve HSPC adhesion to vascular and stromal elements. In this context, Smith-Berdan and colleagues present evidence that short-term VEGF-A preconditioning of recipient mice significantly enhances long-term donor chimerism, suggesting that transient vascular modulation may promote a more receptive BM niche for HSPC engraftment. These findings extend the role of VEGF-A beyond stem cell mobilization, offering new insights into how fine-tuned vascular conditioning might be used to improve HSCT outcomes. Moreover, in patients undergoing high-dose chemotherapy or irradiation for hematologic malignancies, timely recovery of the vascular niche is critical for effective HSC engraftment and hematopoietic reconstitution. Controlled, time-restricted modulation of vascular permeability may therefore serve as a valuable add-on to existing transplantation protocols. Furthermore, this approach may have broader applications in regenerative medicine, where vascular remodeling could facilitate stem cell delivery and tissue repair. Nonetheless, these therapeutic possibilities must be carefully balanced against VEGF’s complex role in disease, as it does not only support physiological regeneration but also contributes to pathological processes, such as extramedullary hematopoiesis and cancer. Future studies will be essential to determine the therapeutic window in which VEGF-driven permeability enhances regeneration without exacerbating disease.

In summary, Forsberg and colleagues demonstrate that tightly regulated vascular permeability can be harnessed to enable safe and efficient HSC mobilization, while also enhancing homing and engraftment (Smith-Berdan et al.). These findings highlight the therapeutic potential of finely timed targeting of the perivascular niches to optimize stem cell trafficking and HSCT outcomes.

## Acknowledgments

Research discussed in this article was funded by an ATRAE Program Award, State Plan for Scientific, Technical and Innovation Research, Ministry of Science, Innovation and Universities, Government of Spain (ATR2023-144970) to S.M.-F. M.J.C. is funded by the Mildred Scheel Fellowship Program for Cancer Research 2025, German Academic Exchange Service (DAAD).

## Declaration of interests

The authors declare no competing interests.

## Declaration of generative AI and AI-assisted technologies in the writing process

During the preparation of this work, the authors did not use AI or AI-assisted technologies in the writing process.
